# Protective Effects of Soluble Collagen during Ultraviolet-A Crosslinking on Enzyme-Mediated Corneal Ectatic Models

**DOI:** 10.1371/journal.pone.0136999

**Published:** 2015-09-01

**Authors:** Xiaokun Wang, Yong Huang, Sabah Jastaneiah, Shoumyo Majumdar, Jin U. Kang, Samuel C. Yiu, Walter Stark, Jennifer H. Elisseeff

**Affiliations:** 1 Wilmer Eye Institute, School of Medicine, Johns Hopkins University, Baltimore, Maryland, United States of America; 2 Department of Electrical and Computer Engineering, Johns Hopkins University, Baltimore, Maryland, United States of America; 3 Ophthalmology, King Khaled Eye Specialist Hospital, Riyadh, Saudi Arabia; 4 Department of Material Science and Engineering, Johns Hopkins University, Baltimore, Maryland, United States of America; 5 Department of Biomedical Engineering, Johns Hopkins University, Baltimore, Maryland, United States of America; Bascom Palmer Eye Institute, University of Miami School of Medicine, UNITED STATES

## Abstract

Collagen crosslinking is a relatively new treatment for structural disorders of corneal ectasia, such as keratoconus. However, there is a lack of animal models of keratoconus, which has been an obstacle for carefully analyzing the mechanisms of crosslinking and evaluating new therapies. In this study, we treated rabbit eyes with collagenase and chondroitinase enzymes to generate *ex vivo* corneal ectatic models that simulate the structural disorder of keratoconus. The models were then used to evaluate the protective effect of soluble collagen in the UVA crosslinking system. After enzyme treatment, the eyes were exposed to riboflavin/UVA crosslinking with and without soluble type I collagen. Corneal morphology, collagen ultrastructure, and thermal stability were evaluated before and after crosslinking. Enzyme treatments resulted in corneal curvature changes, collagen ultrastructural damage, decreased swelling resistance and thermal stability, which are similar to what is observed in keratoconus eyes. UVA crosslinking restored swelling resistance and thermal stability, but ultrastructural damage were found in the crosslinked ectatic corneas. Adding soluble collagen during crosslinking provided ultrastructural protection and further enhanced the swelling resistance. Therefore, UVA crosslinking on the ectatic model mimicked typical clinical treatment for keratoconus, suggesting that this model replicates aspects of human keratoconus and could be used for investigating experimental therapies and treatments prior to translation.

## Introduction

The cornea has a unique matrix structure that is composed of collagen fibrils organized into lamellae and proteoglycans, which intermingle to mediate and provide additional stability to the meta-structure [1]. Keratoconus is an ectatic corneal disorder characterized by progressive corneal axial thinning, corneal protrusion, irregular astigmatism, and decreased vision [2]. Although the cause of this corneal disorder remains unclear, studies suggest that the total amount of protein in keratoconus corneas is decreased, and the level of degradative lysosomal enzymes is elevated in affected areas [3, 4]. Abnormal proteinase, defective enzymatic activity and oxidative damage have also been implicated in keratoconus [5–7]. Early studies reported increased collagenase and gelatinase activities in the medium of keratoconus corneal cultures [6]. Some proteinase inhibitors, including tissue inhibitors of metalloproteinases (TIMPs), α-1-proteinase inhibitor, and α-2-macroglobulin, are decreased in keratoconus [8,9]. Recently, it was reported that the lysosomal proteinases, cathepsin B and cathepsin G were elevated in human keratoconus corneas [4,10,11].

Collagen crosslinking using ultraviolet-A (UVA) irradiation initiated by riboflavin (RF) has recently been developed as a clinical treatment to slow or halt the progression of keratoconus [12]. The crosslinking reactions in the corneas were reported to be carbonyl-based, and mainly occurred within single collagen fibrils and the PG core proteins [13]. In recent years, a large number of studies of collagen crosslinking have shown corneal topographic flattening [14,15], improvement of corrected visual quality [15,16], and reduction of the associated corneal steepening and astigmatic power [17,18]. Thus collagen crosslinking has strong potential to become a standard treatment for keratoconus by slowing the disease’s progression and postponing the need for corneal transplantation.

Unfortunately, there are a number of clinical complications that occur after UVA crosslinking, including a significant reduction of keratocyte density, corneal scarring and corneal tissue thinning [19–21]. The lack of animal preclinical models of keratoconus has been an obstacle for carefully analyzing the mechanisms of crosslinking and evaluating potential new therapies that address the efficacy and complications for the disease and its treatment. Owing to the fact that abnormal collagenase activity and decreased total collagen amount are present in keratoconus, collagenous enzyme exposure to normal tissue may be able to simulate some aspects of acute keratoconus disease.

In this study, we developed a corneal ectatic model that mimics keratoconus by applying collagenase and chondroitinase enzymes to rabbit corneas *ex vivo*. After enzyme treatment, RF-UVA crosslinking was applied to the keratoconus corneas to investigate options for therapeutic optimization, without the aforementioned side effects. Corneal morphology, collagen ultrastructure and thermal stability were evaluated before and after UVA crosslinking. We found that the addition of soluble type I collagen solution during crosslinking prevented extensive cornea thinning in this enzymatic keratoconus tissue model, indicating this treatment has potential as a viable clinical treatment to prevent or reduce complications of conventional UVA crosslinking.

## Materials and Methods

### Tissue Preparation

The animal tissues were purchased from PelFreez Biologicals. Pel-Freez Biologicals produces high quality raw materials and intermediates for biological research and diagnostic manufacturing. The products include lyophilized tissue powders, serum products, plasma, blood complement, tissues, antibodies, and other biological raw materials. Pel-Freez Arkansas has achieved ISO9001: 2008 certification. Certification Registration # is S 951 05 3274. Tissues purchased from the same company were used in many previous publications [13,22].

A total number of 108 albino rabbit eyes (Cat. # 41211–2) immersed in Dulbecco’s Modified Eagle Medium (DMEM) were purchased from Pelfreez Biologicals (Rogers, AR). Samples were separated into nine groups (n = 12 in each group): 1) Untreated (Control); 2) UVA crosslinked control corneas (Control-CXL); 3) UVA crosslinked control corneas with soluble collagen (Control-CXL-Col); 4) Collagenase type II-treated corneas (COLG); 5) UVA crosslinked COLG corneas (COLG-CXL); 6) UVA crosslinked COLG corneas with soluble collagen (COLG-CXL+Col); 7) Chondroitinase ABC (C366710U, Sigma Aldrich) treated corneas (Ch^ase^ABC); 8) UVA crosslinked Ch^ase^ABC corneas (Ch^ase^ABC-CXL); 9) UVA crosslinked Ch^ase^ABC corneas with soluble collagen (Ch^ase^ABC-CXL+Col).

### Enzyme treatment

**Fig 1:**
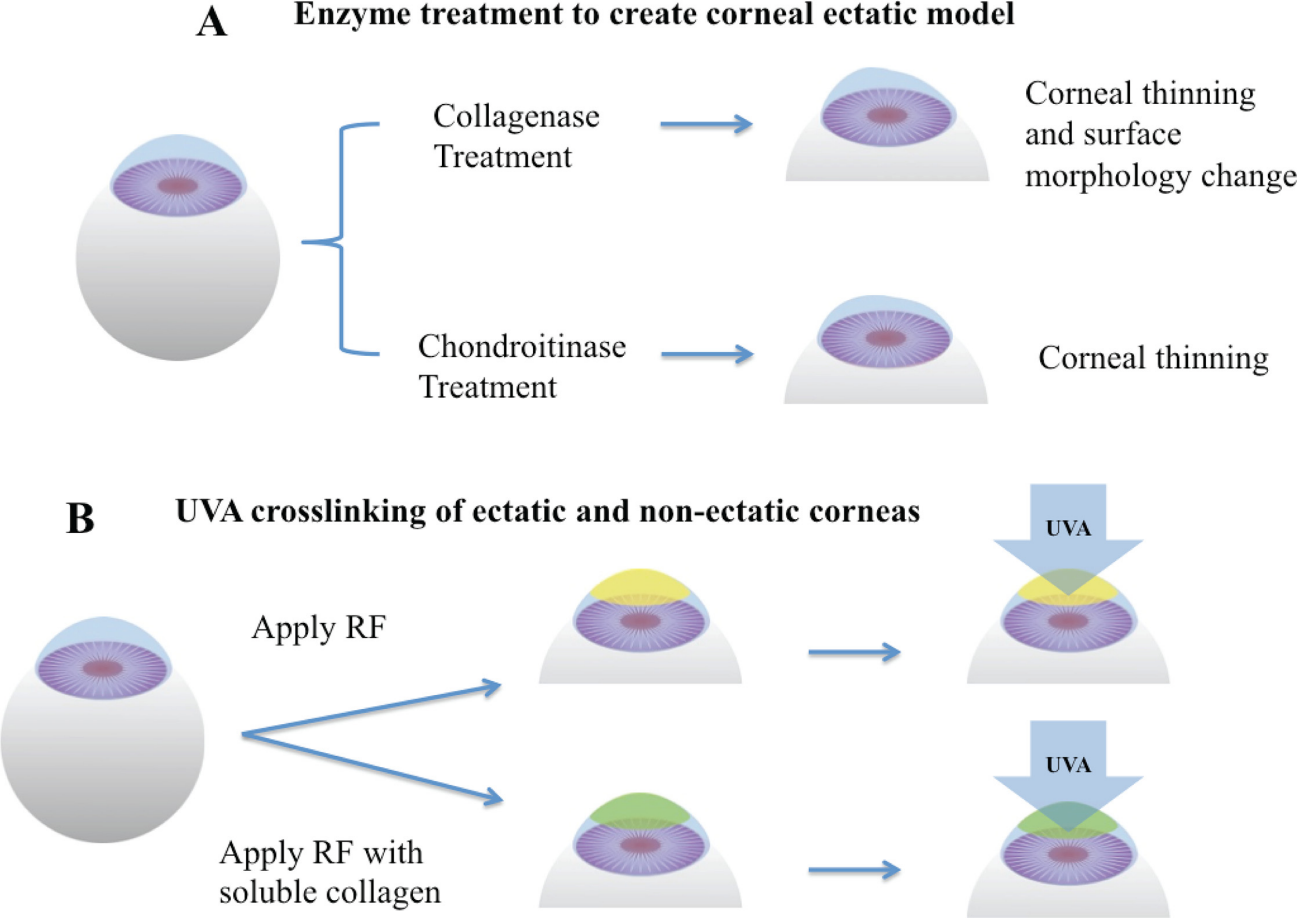
Schematic of enzyme-mediated corneal ectatic model and UVA crosslinking (A) Corneal ectatic model created by collagenase or chondroitinase treatments. (B) UVA crosslinking process with RF and soluble collagen.

The digesting solutions contained 20 U/ml collagenase type II (LS004177 Worthington, 280 U/mg) in PBS, and 0.1 U/ml chondroitinase ABC (C36675U Sigma Aldrich) in Tris buffer, with 50 mM Tris base, 60 mM sodium acetate, and 0.02% bovine serum albumin, pH 8.0. The surfaces of rabbit eyes were wiped with 70% ethanol 15 sec to loosen the epithelial layer. After epithelial removal, rabbit eyes were immersed in enzyme solutions and incubated at 37°C on the shaker at 400 rpm for 3 hours. The treatment was stopped by rinsing in DMEM/10% fetal bovine serum (Gibco) three times. The schematic of the treatment is shown in Fig 1A.

### RF-UVA Crosslinking

Rabbit eyes in UVA CXL groups were soaked in 0.1% riboflavin (RF) dissolved in a 15% dextran-500 (Sigma Aldrich) PBS solution for 20 minutes, and eyes in CXL-Col groups were soaked for 20 minutes in a mixture contained 0.1% RF and 1.0 mg/ml soluble type I collagen (IAC-50 Native collagen from Bovine dermis AteloCell, Koken, Japan) in 15% dextran-500 PBS solution, pH 7.2. After corneas were saturated with riboflavin, samples were placed under a 365-nm UVA light source (F15T8/BL lamp) at 3 mW/cm^2^ for 30 minutes. Light power was measured using a UVA DCS detector (Solar Light Co., Glenside PA). One drop of fresh RF/ or RF+Col solution was applied to the corneal surfaces every 5 minutes to maintain appropriate moisture. After crosslinking, samples were washed in 15% dextran-500 three times for further characterization. The schematic of UVA-CXL is shown in Fig 1B.

Twelve samples in each group were washed in a 15% dextran-500 solution to reduce swelling and prepared for further characterization, including 6 for optical coherence tomography (OCT), and 6 for differential scanning calorimetry (DSC). OCT samples were divided into two sub-groups: 3 eyes in each group were stored in DMEM for 4 hours to serve as “swollen” corneas for OCT, and 3 eyes were stored in 15% dextran-500 for 4 hours to reduce swelling, and the reduced swelling samples were processed for transmission electron microscopy (TEM) after OCT imaging.

### Optical coherence tomography (OCT)

After digestion and crosslinking, 3 eyes in each group were stored in 15% dextran-500 for 4 hours to prevent swelling, and 3 eyes were washed and stored in DMEM for 4 hours to serve as “swollen” corneas. Samples stored in dextran and DMEM were characterized by a real-time 3D OCT system. The real-time 3D OCT system was established in previous studies, which included a line-scan camera (EM4, e2v, USA) with 12-bit depth, 70-kHz line rate, and 2048 pixels as the spectrometer detector, a superluminescent (SLED) light source with an output power of 10 mW and an effective bandwidth of 105 nm centered at 845 nm, which gave an axial resolution of 3.0 μm in air for the experiment. The transversal resolution was approximately 12 μm, assuming a Gaussian beam profile. The scanning area of each cornea had a width of 4.8 mm and depth of 1.2 mm.

### Differential scanning calorimetry (DSC)

The thermal stability of the corneas was assessed with DSC 8000 calorimeter (Perkin Elmer, Waltham MA) over a temperature range of 10°C to 95°C. Corneas were dissected from the eyeballs and dried with delicate task wipers (Kimwipes, KIMTECH Science) to remove excess dextran. Three samples of each group were punctured using a 6 mm biopsy punch, then placed in 50 μL aluminum pans and crimp-sealed. An empty pan was used as reference in all samples. DSC was performed at a rate of 5°C per minute under a 20 mL/min nitrogen flow. Transition temperatures were detected and analyzed with the Pyris software (Perkin Elmer) version 10.1 for Windows and exported to a spreadsheet.

### Transmission electron microscopy (TEM)

After enzyme treatment and crosslinking, samples stored in dextran 500 were fixed in 3% paraformaldehyde (freshly prepared from EM grade pill form), 1.5% glutaraldehyde, 5 mM MgCl_2_, 5 mM CaCl_2_, 2.5% sucrose, 0.1% tannic acid in 0.1 M sodium cacodylate buffer, at pH 7.2, and stored overnight at 4°C. After the buffer rinse, samples were post-fixed in 1% osmium tetroxide (1 hour) on ice in the dark, followed by deionized H_2_O rinse. Samples were stained with 2% aqueous uranyl acetate (0.22 μm filtered, 1 hr, in dark) and dehydrated through treatment in increasingly concentrated ethanol (from 30% to 100%). After 70% ethanol dehydration, the anterior half (around 150~200 μm) of each cornea was cut for further dehydration and embedding in Eponate 12 (Ted Pella, Redding CA) resin. Resin blocks were polymerized for two to three days at 37°C before transferring to 60°C overnight. Thin sections, from 60 to 90 nm, were cut at a depth of 50 μm from the surface with a diamond knife on the Reichert-Jung Ultracut E ultramicrotome and picked up with naked copper grids. Grids were stained with 2% uranyl acetate in 50% methanol and observed with Philips/FEI BioTwin CM120 Transmission Electron Microscope at 80kV. Images were captured with an AMT CCD (1K x 1K) camera.

### Image analysis

Image analysis was carried using MATLAB. Original images were binarized using an adaptive thresholding method. In order to remove noise, a morphological operator (opening) was applied to the binary images. For each connected component in the binary image, eccentricity was measured. Those components with large eccentricity were excluded for further analysis. To estimate the density of fibers, a window of fixed size (300x300 dpi) was randomly localized. Within the window, the density (i.e. the number of fibers per unit area) was measured and recorded. This random procedure was repeated 200 times and the density of fibers was estimated using the median number.

### Statistical Analysis

Student’s *t*-test was used to statistically examine differences between normal corneas, ectatic corneas, and cross-linked corneas with or without soluble collagen in terms of corneal thickness, collagen fibril diameter, interfibrillar spacing, density of fibril, and transition temperature. The independent samples *t*-test statistical analysis was performed pairwise between the enzyme-treated groups and untreated control group, and between the crosslinked groups and non-crosslinked ones with the same enzyme treatment. A probability of P<0.05 was taken to indicate a statistically significant difference.

## Results

### OCT Characterization of Ectatic and Crosslinked Corneas

**Fig 2:**
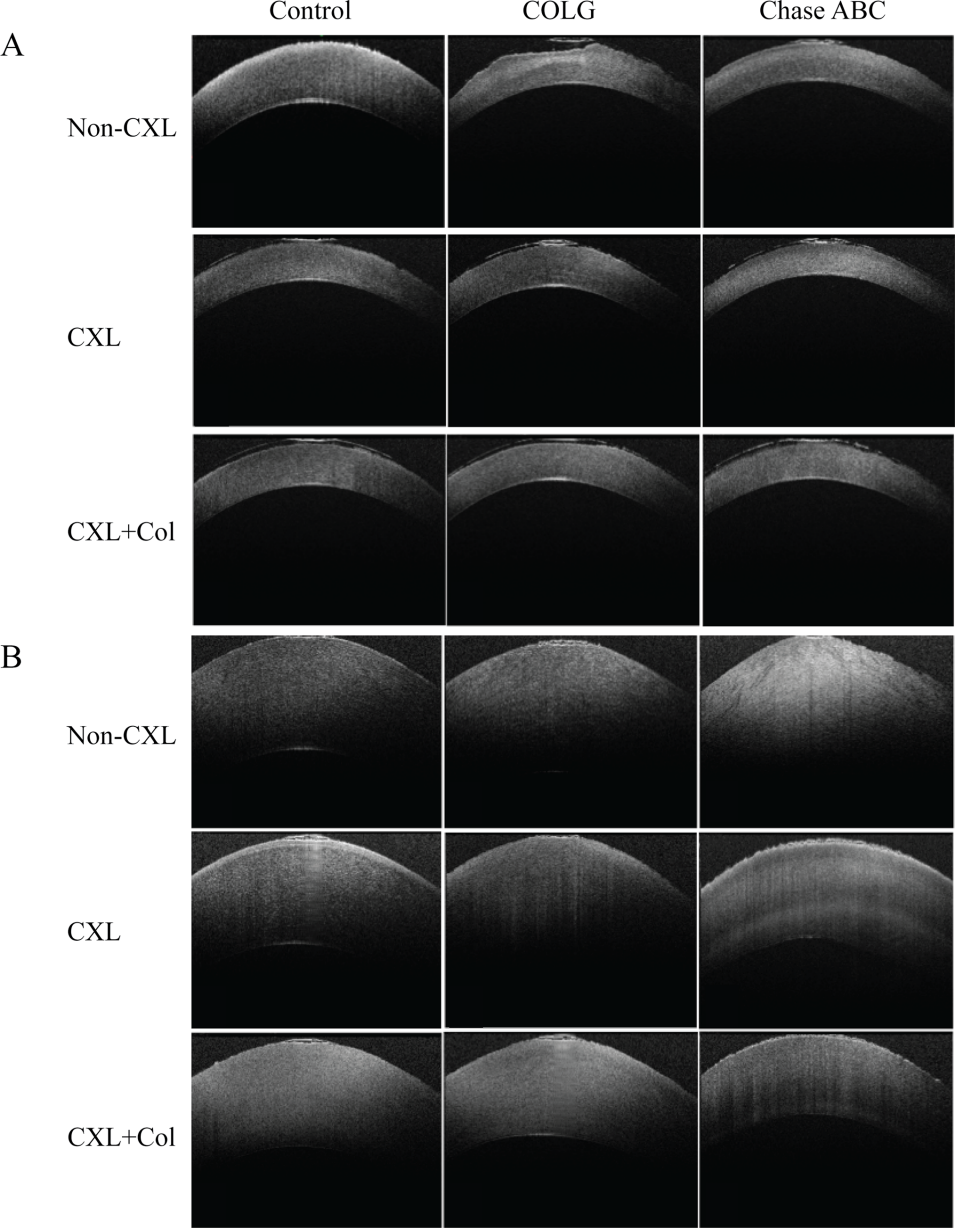
The optical coherence tomography (OCT) aspects of different corneal ectatic models after corsslinking and swelling. (A) Corneas were preserved in 15% dextran, and (B) Corneas were preserved in DMEM for 4 hours.

Enzyme treatments of rabbit corneas *ex vivo* resulted in corneal surface erosion and corneal thinning, as characterized by OCT (Fig 2A). The mean corneal center thickness (CCT) before and after enzyme treatment and crosslinking are listed in Table 1. The control group had a thickness of 362±12.0 μm. COLG treatment resulted in corneal thinning to 331±10.5 μm and surface erosion, and Ch^ase^ABC treatment decreased the corneal thickness to 316±6.5 μm. UVA crosslinking resulted in corneal thinning in all samples compared with Non-CXL ones. CXL and CXL+Col smoothened the corneal surface. In all the CXL+Col groups, the average central corneal thickness (CCT) was slightly greater in comparison to the conventional CXL samples in the same treatment group. As for the swelling groups (Fig 2B), enzyme treatments decreased the swelling resistance of corneas. Control corneas after swelling in DMEM for 4 hours had an average thickness of 718±7.0 μm (Table 1); COLG treatment increased the swelled thickness to 791±9.5 μm, and Ch^ase^ABC treatment resulted in a swelled thickness of 844±12.5 μm. UVA CXL restored the resistance of swelling in both control and ectatic corneas. Furthermore, the CXL+Col groups reduced swelling by 6–17% in comparison to the conventional CXL corneas in the same enzyme treatment group. There was minimal change among the control groups, as crosslinking and treatment with soluble collagen reduced swelling by less then 4%.

**Table 1:** Central Corneal Thickness (CCT) of rabbit corneas before and after enzyme treatment and UVA crosslinking.

	Central corneal thickness (μm)	Swelled Central corneal thickness (μm)
Control	362±12.0	718±7.0
Control-CXL	348±7.5	683±8.5 *
Control-CXL+Col	355±6.5	678±6.0 *
COLG	331±10.5 *	791±9.5 *
COLG-CXL	325±5.0	765±9.0
COLG-CXL+Col	338±8.5	722±7.5 #
Ch^ase^ABC	316±6.5 *	844±12.5 *
Ch^ase^ABC-CXL	235±13.0 ^	663±10.5 ^
Ch^ase^ABC-CXL+Col	265±10.0 ^	552±11.0 ^

Values denote mean ± standard deviation

*p<0.05 compared with the Control corneas

#p<0.05 compared with the COLG corneas

^p<0.05 compared with the Ch^ase^ABC corneas

### Ultrastructural Characterization of Ectatic and Crosslinked Corneas

**Fig 3:**
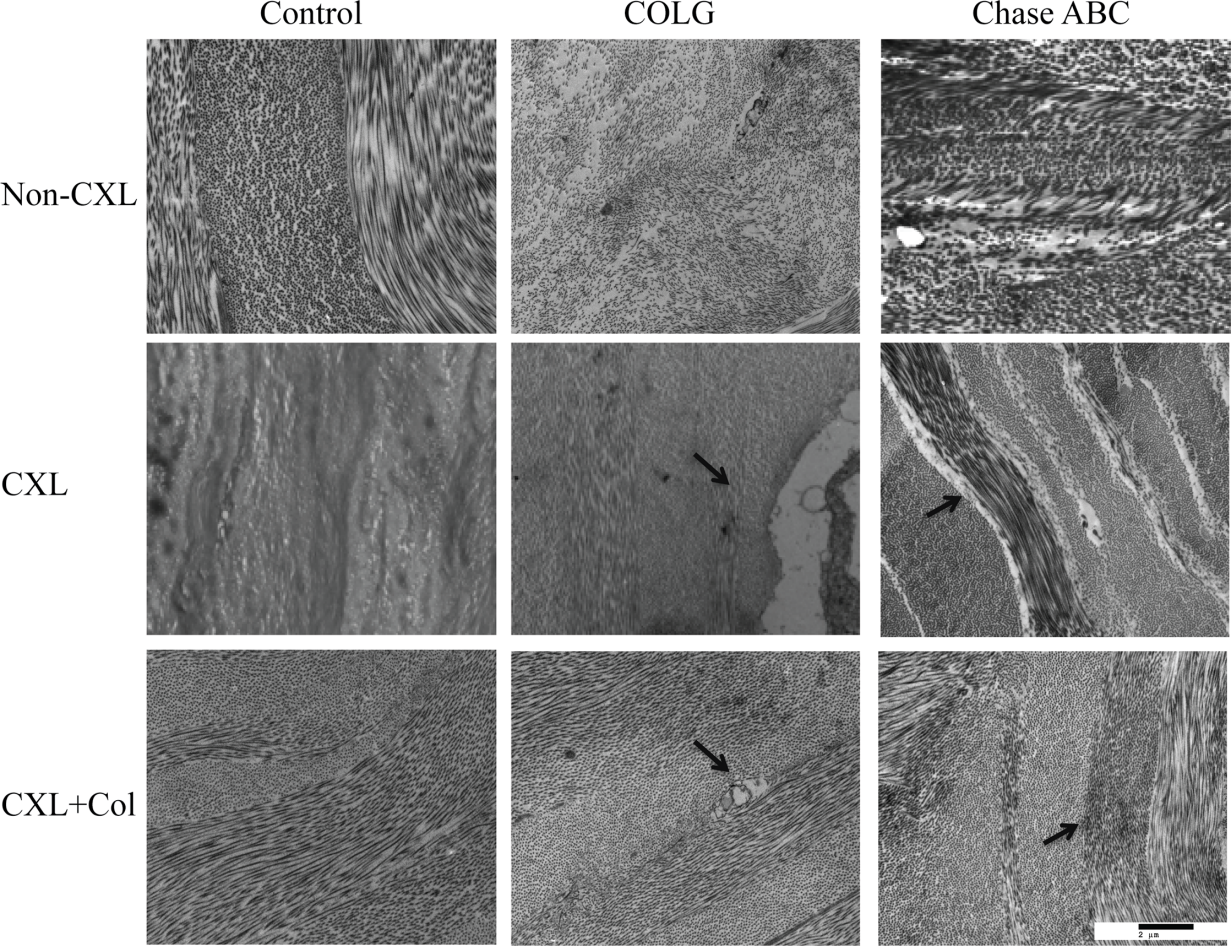
Transmission electron microscope images of corneal ectatic model before and after crosslinking. Ultrastructure of control rabbit cornea under 9700x magnification. Column from left to right: Control cornea, COLG treated cornea, and Ch^ase^ABC treated cornea. Row from top to bottom: Non-CXL, CXL and CXL+ collagen

Ectatic corneas exposed to COLG or Ch^ase^ABC demonstrated collagen ultrastuctural damage in the stroma (Fig 3). In comparison to control corneas, COLG and Ch^ase^ABC treatments led to a decrease in fibril density, similar to keratoconus. After crosslinking, Control-CXL showed an increase of fibril density and lamellae condensing compared to non-CXL group, and Control-CXL+Col had a similar effect to the Control-CXL group. In the ectatic corneas, UVA crosslinking revealed ultrastructural damages. In the COLG-CXL group, although UVA crosslinking increased the fibril density in the stroma, large tears between the collagen lamellae and keratocytes were noted. In the Ch^ase^ABC-CXL group, gaps between lamellae existed after UVA crosslinking. When soluble collagen was added in the crosslinking system, no tear between keratocytes and lamellae in the COLG-CXL+Col group, and no gaps between the lamellae in the Ch^ase^ABC-CXL+Col group were observed, suggesting a protective effect of collagen. The overall morphologies of the collagen ultrastructures in the CXL+Col groups were similar to the uncrosslinked control corneas.

**Fig 4:**
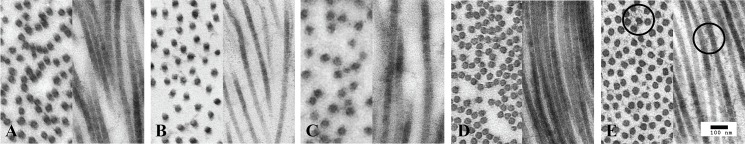
Transmission electron microscope images of corneal ectatic model. (A) Control cornea; (B) COLG treated cornea, and (C) ChaseABC ABC treated cornea. (D) ChaseABC treated cornea crosslinked without collagen, (E) ChaseABC treated cornea crosslinked with collagen.

High-magnification TEM images of enzyme-treated and crosslinked Ch^ase^ABC corneas are shown in Fig 4. In comparison to control cornea (Fig 4A), COLG group resulted in decrease of the fibril diameter and fibril density (Fig 4B), and damaged characteristic D-banding patterns of collagen fibril were observed in Ch^ase^ABC group (Fig 4C). Ch^ase^ABC-CXL group (Fig 4D) had a significantly higher fibril density as compared to Ch^ase^ABC group (Fig 4C). Ch^ase^ABC-CXL+Col group (Fig 4E) revealed less condensing of the fibrils in comparison to Ch^ase^ABC-CXL group, and additional staining of collagen in between the fibrils, which could possibly be the crosslinked soluble collagen. Fiber diameter, interfibrillar spacing and fibril density changes before and after enzyme treatments and crosslinking are shown in Table 2. Average collagen fibril diameter decreased in both COLG and Ch^ase^ABC groups, and Ch^ase^ABC resulted in larger ranges of fiber diameter distribution. Interfibrillar spacing was characterized by analysis of the distance between each spot, and its closest neighbor on the image. Compared with the control group, both COLG and Ch^ase^ABC groups had wider interfibrillar spacing and significantly decreased fibril density. Crosslinking with or without soluble collagen resulted in a significantly decrease of interfibrillar spacing, and an increase of fibril diameter and density.

**Table 2:** Fibril diameter, interfibrillar distance and density of spot of rabbit corneas before and after enzyme treatment and UVA crosslinking.

	Fibril diameter (nm)	Distance between nearest spot (nm)	Density of spot
Control	40.67±0.92	72.3±0.89	18.0±0.47
Control-CXL	42.38±0.62 *	69.4±2.01 *	24.6±0.53 *
Control-CXL+Col	40.82±0.53	58.7±2.81 *	25.5±0.92 *
COLG	36.37±2.15 *	75.4±1.22 *	13.9±1.56 *
COLG-CXL	37.21±3.44 #	62.4±2.78 #	23.5±1.38 #
COLG-CXL+Col	37.03±2.18 #	60.7±3.22 #	24.2±1.42 #
Ch^ase^ABC	40.45±3.81	78.8±4.00 *	13.1±0.97 *
Ch^ase^ABC-CXL	41.55±2.02 ^	63.8±4.67 ^	25.0±2.06 ^
Ch^ase^ABC-CXL+Col	41.57±1.53 ^	61.8±4.13 ^	25.7±3.22 ^

Values denote mean ± standard deviation

*p<0.05 compared with the Control corneas

#p<0.05 compared with the COLG cornea

^p<0.05 compared with the Ch^ase^ABC corneas

### Thermal Stability

**Fig 5:**
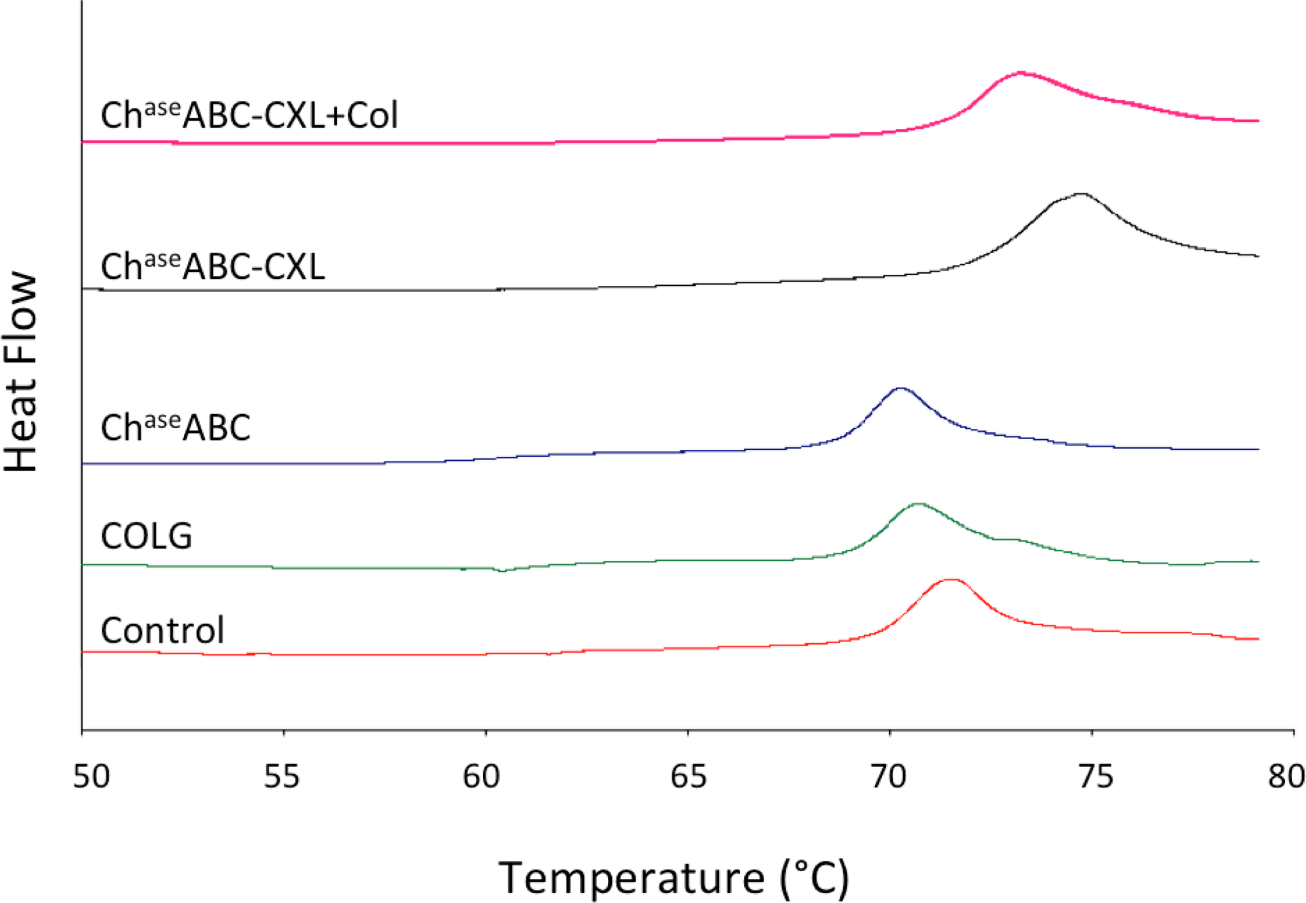
Differential scanning calorimetry thermograms of ectatic corneas before and after crosslinking.

The DSC thermograms highlighted the matrix thermal stability changes after enzyme treatments (Fig 5). All samples presented one peak, which related to the temperature of thermal denaturation of collagen. After degradation, the transit temperature of COLG group and the Ch^ase^ABC group shifted to the lower temperature range (Table 3). After UVA crosslinking (with or without soluble collagen), the transition temperature increased as compared to the corneas with the same treatment.

**Table 3:** Transition temperature of rabbit corneas before and after enzyme treatment and UVA crosslinking

	Transition temperature (°C)
Control	71.99±0.77
Control-CXL	74.55±0.25 *
Control-CXL+Col	73.84±0.72 *
COLG	71.22±0.20
COLG-CXL	74.31±0.53 #
COLG-CXL+Col	73.96±0.65 #
Ch^ase^ABC	70.70±0.68
Ch^ase^ABC-CXL	74.95±0.72 ^
Ch^ase^ABC-CXL+Col	73.68±0.67 ^

Values denote mean ± standard deviation

*p<0.05 compared with the Control corneas

#p<0.05 compared with the COLG corneas

^p<0.05 compared with the Ch^ase^ABC corneas

## Discussion

**Fig 6:**
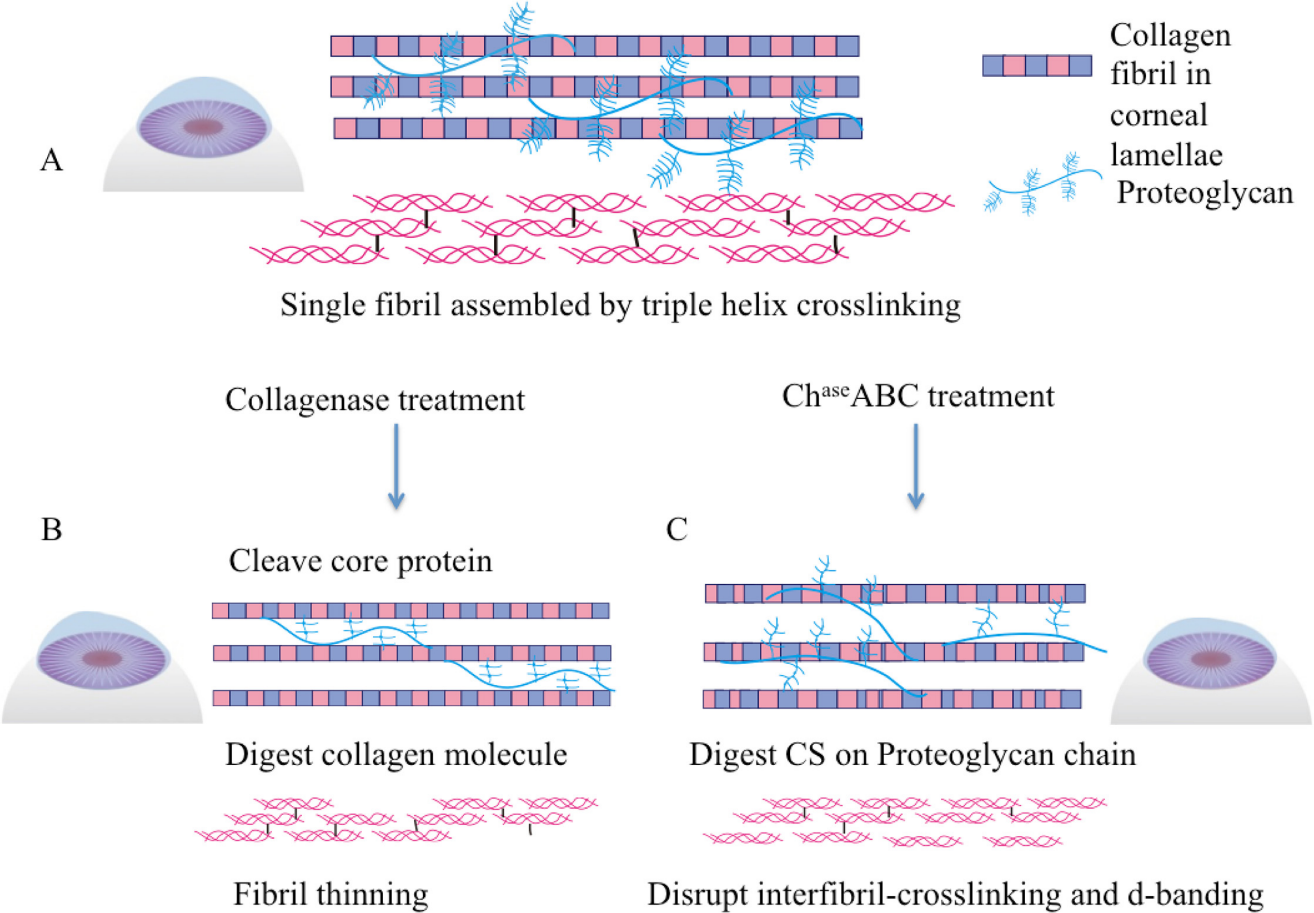
Simplified mechanism of the enzyme treatment to collagen fibrils. (A) In healthy corneas, proteoglycans plays an important role of intrafibril interaction. (B) Collagenase treatment degraded protelglycan core protein, as well as collagen helix molecules in the fibril, which resulted in fibril diameter decrease. (C) Chondroitinase treatment degraded the glycosaminoglycan, which resulted in weakened intrafibril crosslinking, and damaged single fibril structure.

Keratoconus is a disease that can cause serious vision loss and necessitate corneal transplantation as it progresses. UVA crosslinking provides an approach to slow or even halt the progression of keratoconus, and restore vision [23]. However, there have been reports of complications with the UVA crosslinking approach, including stromal scaring [24,25], endothelial cell loss [26], and corneal melting [27,28]. The complication rate of crosslinking, defined as the percentage of eyes losing two or more Snellen lines, is 2.9%; the failure rate (percentage of eyes with continued progression), by contrast, is 7.6% [29], which indicates that the crosslinking approach needs optimization to avoid failure and complications. Unfortunately, owing to the lack of readily available ex vivo tissue models of keratoconus, systematical studies of mechanisms and potential improvements of UVA crosslinking have been difficult. The main clinical feature of keratoconus is thinning and ectasia of the cornea [21,29,30]. These features have usually been associated with the degradation of the extracellular matrix caused by abnormal matrix metalloproteinase activity [31,32]. Enzymatic malfunction may relate to the ultrastructural change of collagen fibrils[33], decreases in corneal mechanical strength [34,35] and the characteristic cone-shaped textures [36–38]. In this study, we developed an ex vivo rabbit corneal ectatic model that mimics aspects of keratoconus. Rabbit is the most commonly used animal for corneal research [39]. Although rabbit corneas don’t have the thin (8~12 μm) Bowman’s layer that is found in human corneas [40], they are suited for ophthalmological research for several reasons. Rabbit corneas have similar size, curvature and comparable stromal thickness to human corneas [41]. Previous 3D microanatomy comparisons of the rabbit and human cornea structure found that the 3D organization of the stromal lamellae was similar in both species [42]. Specifically for UVA crosslinking studies, many preclinical studies employed the rabbit model in attempts to understand the mechanism and effectiveness of collagen crosslinking [22]. We treated rabbit cornea explants with collagenase type II and chondroitinase ABC to create an acute disease model to mimic the dysfunctional corneal structures, and investigate the protective effect of adding soluble collagen solution during UVA crosslinking. The simplified mechanism of collagenase and chondroitinase treatment is indicated in Fig 6. Collagenase type II used in this study mostly degraded the proteoglycan core proteins without causing drastic damages of corneal stroma that predominantly contains type I collagen [43]. Chondroitinase ABC was used to degrade chondroitin sulfate, which is one of the major components of proteoglycans in corneas [44].

The central corneal thickness (CCT) of New Zealand white rabbits characterized *in vivo* by OCT was 373 ± 7.2 μm [45]. In our study, rabbit eyes stored in 15% dextran-500 had the CCT approximately 350 μm, which is within a reasonable range for normal rabbit corneas. Both enzyme treatments resulted in corneal thinning, which is the major symptom of keratoconus [46,47], and caused corneal surface erosion likely due to the damage of proteoglycan core proteins and glycosaminoglycan. Proteoglycans play an important role in the collagen interfibrillar crosslinking and intrafibrillar interaction and spacing [46], and the loss of their function will result in morphological changes and hydrodynamic malfunction, such as a decrease in resistance to stromal swelling. The swelling effect of collagenase treated samples was also reported by Hong *et al*. [48]. In addition, a proteoglycan-deficient study by Chakravarti *et al*. [49] reported that lumican-deficient posterior stroma displayed increased fibril diameter and large fibril aggregates. These data could explain why the Ch^ase^ABC treatment resulted in a larger range of fiber diameters, less defined fibril edges and the partial damage of the D-bandings.

In keratoconus patients, collagen ultrastructural changes vary from case to case. For instance, Patey *et al*.[50] studied 31 cases of human keratoconus by image analysis, and reported collagen fibril diameter slightly increased in the keratoconus corneas (225 Å compared to 216 Å) and interfibrillar distance also increased with disease (445 Å compared to 435 Å). A more recent ultrastructural study by Akhtar *et al*. [32,51] analyzed 3 normal corneas and 3 keratoconus corneas, and showed collagen fibril thinning and increase in interfibrillar spacing in one case, and a slight decreased inter-fibrillar spacing in the posterior stroma of another. In our study, our enzyme-mediated acute ectatic model could represent partially of the keratoconus symptoms, including the decrease in corneal thickness, collagen fibril thinning and changes in interfibrillar spacing.

**Fig 7:**
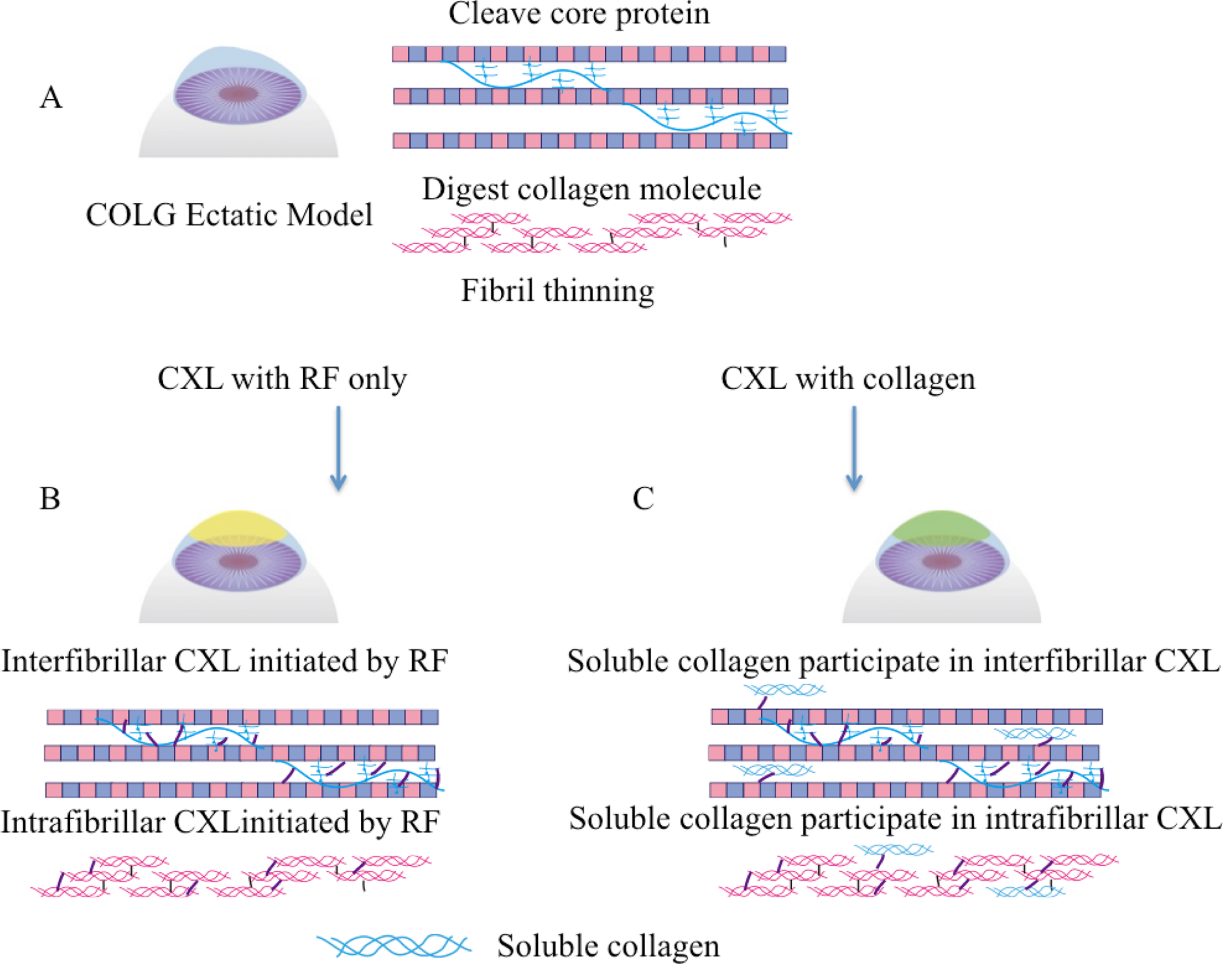
Simplified mechanism of collagen crosslinking with and without soluble collagen in collagenase treated corneas. (A) Ectatic corneas with ultrastructural damages, (B) Crosslinking without soluble collagen happens between proteoglycan and collagen fibrils, and also between collagen molecules, and proteoglycan interfigril; (C) Crosslinking with soluble collagen happens not only between native collagen fibril and proteoglycans, but also between soluble collagen and native collagen, and soluble collagen and proteoglycan.

The success of riboflavin/UVA crosslinking to reduce keratoconus progression has been well documented [52,53], so have the complications [20,23,54]. The possible crosslinking reaction in corneas happens between collagen molecule and proteoglycan, inter-proteoglycan, and intra-proteoglycan [55]. In our crosslinking system, we added soluble type I collagen as a potential protective reagent extracellular matrix supplement to the ectatic corneas. Type I collagen usually has a molecular weight around 300kDa, and this large molecule will be difficult to penetrate epithelium layer. Previous studies of corneal organ culture have proved that large molecules such as dextran-500 (Mw-450~600 kDa) [56] are able to penetrate into the stromal extracellular space even with the epithelium and endothelium layer intact. Based on these results, we hypothesize that soluble type I collagen will also penetrate into the anterior stroma with epithelium removal, and participate the crosslinking. The hypothesized mechanism of RF/UVA crosslinking with the participation of soluble type I collagen is depicted in Fig 7. Crosslinking is known to stabilize the collagen structure [57,58], as evidenced in our study, all CXL groups regained swelling resistance and thermal stability. UVA crosslinking of the control corneas increased fiber diameter and density, similar to previous reports by Akhtar *et al*. [32] and Wollensak *et al*. [59]. However, crosslinking of ectatic corneas revealed potential ultrastructural damages. The gaps between lamellae in the Ch^ase^ABC-CXL group and the damage to keratocyte in COLG-CXL group provided evidence that UVA irradiation causes more severe damage to ectatic corneas as compared to control corneas. UVA irradiation generates reactive oxygen species which also raise the risk of photochemical damage to corneal tissue [60]. These type of injuries in the ectatic corneas could cause a wound-healing response, involving keratocyte apoptosis, proliferation, and migration [61], which may lead to corneal scarring. In our study, by supplementing soluble collagen to the ectatic corneas that have protein and proteoglycan dysfunctions during crosslinking, CXL-Col groups presented no ultrastructural damages after crosslinking and a better swelling resistance. These data indicated that the free soluble collagen molecules strengthen the corneal collagen fibrils possibly by crosslinking with the fibril and/or proteoglycans, and preventing intensive fibril condensing. Soluble collagen could therefore be added to keratoconus eyes prevent any adverse effects during crosslinking.

Previous study of collagen type I from different species reported the transition temperatures ranging from 50~90°C [62]. In our study, the transition temperature of control rabbit corneas was around 71°C. Within the temperature range from 10°C to 95°C, one broad peak appears for each sample, which is associated with the helix-coil transition induced by the thermal disruption of hydrogen bond that stabilize the triple helical collagen structure. Decreased transition temperature in collagenase and chondroitinase treated corneas indicates the weakening of the hydrogen bonds that stabilize the triple helical structure. The significant increase in the transition temperature after crosslinking with or without soluble collagen indicates restored cornea stability.

## Conclusion

In this study, we generated an ex vivo corneal ectatic model that simulates the morphology and structural disorder of keratoconus. This model was then employed to evaluate the protective effect of soluble collagen during UVA crosslinking of on ectatic corneas compared to healthy controls. The corneal ectatic model produced changes in corneal curvature, damage in ultrastructure, and a decrease in thermal stability, similar to the human keratoconus disease. UVA crosslinking restored corneal swelling resistance and thermal stability of the ectatic corneas. Crosslinking with soluble type I collagen solution protected the ectatic corneas against ultrastructural damage caused by conventional crosslinking. This finding indicates soluble collagen will benefit the ectatic corneas in terms of thermal and structural stability. With this new model, the crosslinking procedure and other experimental treatments could be further optimized for eventual translation to patients.
